# Design of a public health emergency equipment modularized system for bioterrorism events in major public places based on scenario analysis, literature review, cluster analysis, and Delphi consultation

**DOI:** 10.3389/fpubh.2025.1513319

**Published:** 2025-04-17

**Authors:** Xiaowen Sun, Yongzhong Zhang, Chao Zhao, Tiantian Li, Song Bai

**Affiliations:** ^1^School of Disaster and Emergency Medicine, Tianjin University, Tianjin, China; ^2^Center for Biosafety Research and Strategy, Tianjin University, Tianjin, China; ^3^School of Chemical Engineering and Technology, Tianjin University, Tianjin, China

**Keywords:** bioterrorism, health emergency, scenario analysis, equipment system, modularization

## Abstract

**Objective:**

To improve the standardization of health emergency equipment at the scene of bioterrorism events in major public places and to establish a health emergency equipment modularized system for such events.

**Methods:**

Based on domestic laws and regulations, domestic and international literature on health emergencies from 2004 to 2023, and survey data on the equipment configuration of domestic health emergency equipment enterprises and relevant emergency response teams, this study first used scenario analysis to explore the functional requirements of health emergency equipment from three aspects: the environment in which bioterrorism events occur, the survival and task requirements of personnel at the scene, and the methods of emergency response. Second, with reference to national standards for the allocation of health emergency equipment, a preliminary plan for the equipment system was determined through literature analysis and market research. Then, a preliminary plan of the modularized system of health emergency equipment is formed through the cluster analysis method. Finally, the Delphi method was used to revise and improve the plan.

**Results:**

The positive coefficient of expert consultation in both rounds was 100%, and the mean coefficient of expert authority was 0.855. The coordination coefficients of the expert opinions were 0.226 and 0.258, respectively (both *P* < 0.001), indicating a good degree of coordination among the expert opinions. The final modularized system of health emergency equipment for bioterrorism events in major public places was established. This modularized system consisted of 5 first-level modules, 15 second-level modules, and 32 third-level modules, and is equipped 162 types of equipment.

**Conclusion:**

The constructed modularized system of health emergency equipment for bioterrorism events has a certain degree of pertinence and reliability and can serve as a reference for the construction of health emergency team equipment modularized systems and the use of health emergency equipment at the scene of bioterrorism events.

## 1 Introduction

In October 2001, several letters containing *Bacillus anthracis* spores brought “bioterrorism” to the forefront of international attention (1). Although bioterrorism events in major public places are infrequent, when they occur, they can cause great losses to lives and property for the State and the people, resulting in serious consequences such as social panic (2). There are still many issues that need to be researched on how to effectively respond to a bioterrorism event, and the modularization of health emergency equipment is one of the important ones. Health emergency equipment is the material basis for an effective response to health emergencies, and the level of effectiveness of the health emergency equipment system determines whether the health emergency work can be carried out smoothly and efficiently.

After the anthrax mail incident in 2001, the U.S. government attached great importance to the construction of the health emergency equipment system, forming a reserve system of basic research, national procurement, and systematic support. The National Pharmaceutical Stockpile specializes in the research and development of medicines, vaccines, and medical equipment to deal with bioterrorism, and the research chain of medicines and vaccines is complete, with a large amount of investment, so that at present there are vaccines and medicines for the prevention and treatment of many bioterrorism agents (3). The US Army Edgewood Chemical Biological Center has also conducted research and development on biological detection, early warning devices and other equipment (4). The equipment of the United States is constantly updated and upgraded with a high level of intelligence and information technology, which improves the efficiency and accuracy of emergency response.

In 2008, in order to strengthen the construction of health emergency teams, accelerate the realization of the standardization and standardized management of health emergency equipment, and improve the capacity of health emergency, the former Office of the Ministry of Health of China issued a circular on the issuance of the “*Health Emergency Response Teams Equipment Reference Catalog (Trial)*” (5), which requires that all parts of the country combine the actual situation, summarize the experience and lessons learned from the successive health emergency response and rescue operations, and attach full importance to the construction of health emergency response teams' equipment. Since 2014, China's emergency team has actively participated in international and domestic assistance on several occasions (6–8), and during the assistance process, China's health emergency equipment has been tested and upgraded, providing valuable experience for the development of China's health emergency equipment system.

After the COVID-19, the country paid more attention to the improvement of the health emergency equipment system, unlike the previous promulgation of the “Emergency Regulations for Public Health Emergencies” and other regulations for traditional emergencies, in 2020 China promulgated the “Biosafety Law of the People's Republic of China” (9), marking the bioterrorism prevention and control has become an important part of China's efforts to establish a national security system (10). This puts a higher demand on the construction of health emergency equipment system for bioterrorism. Compared with the construction of health emergency response equipment systems for conventional emergencies such as earthquakes, floods, and traffic accidents, the modularization of health emergency equipment systems for bioterrorism event has yet to be further studied. Therefore, an important issue is how to scientifically and reasonably equip health emergency equipment and develop a standardized and modularized health emergency equipment system for on-site health emergencies involving bioterrorism events in major public places. This study applies the scenario analysis, literature analysis and market research, and the Delphi method to explore the construction of modularized system of health emergency equipment for bioterrorism events in public places in China.

## 2 Materials and methods

### 2.1 Materials

Refer to the current laws and regulation document on health emergency or health emergency equipment (5, 11–15), such as *Health Emergency Response Team Equipment Reference Catalog (Trial)* (5), *Classification Catalog of Key Materials for Emergency Support (2015)* (15), etc. In the Web of Science, Ei CompendexWeb, PubMed, CNKI, and Wanfang databases, to search the literature related to bioterrorism health emergency equipment published at home and abroad from January 1, 2004 to December 31, 2023, which published at home and abroad from January 1, 2004 to December 31, 2023. The search strategies utilized a combination of the following terms: “bioterrorism,” “bioterrorism event,” “bioterrorism attack,” “health emergency,” “equipment,” “module,” and “modularized system.” And a total of 84 documents were obtained after the screening and extraction of the information. At the same time, more than 10 relevant academic works have been collected, such as *Emergency Medical Rescue Equipment Science* (16), *Emergency Medical Rescue and Equipment Support* (17), etc.

### 2.2 Methods

#### 2.2.1 Scenario analysis

Scenario analysis, also known as scenario description and scenario construction, is a method of analyzing future developments and describing a wide range of possible future outcomes. The advantage of this method is the ability to analyze long-term uncertainty scenarios as well as the lack of data and non-quantitative factors (18). This study applies scenario analysis to determine health emergency equipment functional needs in a realistic scenario of a major public bioterrorism events and discusses the functional needs of bioterrorism emergency equipment from three aspects: the environment at the time of the events, the needs of personnel at the scene, and the methodological approach adopted in the emergency response to a bioterrorism events.

#### 2.2.2 Literature analysis and market research

Analyzing the existing literature can quickly, efficiently, and relatively scientifically soundly identify health emergency response equipment suitable for use at a bioterrorism site. Referring to the relevant laws and regulations on health emergency in China as well as searching the academic research results in the field of health emergency equipment at home and abroad from January 1, 2004 to December 31, 2023, and sort out and summarize the equipment equipped and used in all kinds of emergency health work. Based on the *Practical Guide to Emergency Products in China* (19–21) published by the Operation Monitoring and Coordination Bureau of China's Ministry of Industry and Information Technology and the China Small and Medium Enterprises Development and Promotion Center in 2015, we conducted a research on the types, functions, and parameters of the domestic sanitary and emergency response equipment through phone calls, emails, and field research. At the same time, a survey has been conducted on the equipment configuration of military and local nuclear and biochemical emergency rescue teams and teams for the prevention and control of sudden-onset acute infectious diseases. The above literature analysis and research results provide an important basis for the construction of the modularized system of health emergency equipment in major public places.

#### 2.2.3 Build a modularized system of health emergency equipment

Modularization is the process of establishing a modular system using decomposition and combination methods from a system viewpoint (22). The modular process includes module division and module reorganization.

##### 2.2.3.1 Module division

Cluster analysis is a common data mining process that identifies latent patterns and groupings in a dataset (23). Cluster analysis is classified according to the internal connection rules of the properties of things according to the clustering rules, and the aggregated data can be regarded as a module (24). Therefore, cluster analysis is an effective mathematical modeling approach for module division (25).

Using cluster analysis method for module division, each equipment item should be numerical value for mathematical calculation, Design Structure Matrix (DSM) was used to realize the numerical and matrix of each equipment (26). The DSM is an N item (n_*ij*_) represents a relationship between two elements (i, j), and the number size indicates the strength of the relationship between the elements (27).

First, the 32 equipment functional units are numbered in turn (N1–N32), and then the relationship matrix is determined according to the interrelationship of each equipment functional unit. The relationship between two equipment functional units can be determined according to whether the specific functions of each unit have the same or a cooperative relationship. If they have the same or a cooperative relationship, each item in the matrix is assigned a value of “1”, if not, a value of “0”.

The euclidean distance is used for clustering operations to calculate the absolute distance between different samples and measure their similarity (28). Denoting the euclidean distance between two points by *d*, shown as Equation 1 (29):


(1) $$\begin{array}{l} d\left(n_{i},n_{j}\right)=\sqrt{\sum_{i\neq j,i,j=1}^{n}{\left(n_{i}-n_{j}\right)}^{2}} \end{array}$$

##### 2.2.3.2 Module reorganization

Module reorganization based on the relationships between different modules, so as to realize the hierarchical and structural relationships between modules. Module reorganization can also refer to the functional cooperation and order relationship of each module.

#### 2.2.4 Delphi consultation

The Delphi method was applied to revise and complete the modular system of on-site health emergency equipment for bioterrorism events in major public places.

##### 2.2.4.1 Expert selection

Fourteen experts with experience in biosafety and health emergency response were invited from national and local centers for disease control and prevention, colleges and universities, research institutes, and relevant units of the military to participate in the expert consultation. Table 3 shows the demographics of the 14 experts (30).

##### 2.2.4.2 Design of the consulting questionnaires

The questionnaire was composed of four major parts. The first part was letters to the experts, including an introduction to the research content and purpose as well as notes. The second part included demographic information and the degree of authority of the experts [including the basis of expert judgment (*Ca*) and familiarity with the question (*Cs*)]. The third part included a consultation questionnaire on the modularized system framework for health emergency equipment for bioterrorism events in major public places, which was rated on a Likert scale (1 = not rational, 5 = very rational) (31). A Likert scale is used to judge whether the modules are reasonable or not, and expert opinion columns are set up in each module for the experts to propose modifications; and the fourth part was a health emergency equipment allocation table. For each type of equipment, two options of agree and disagree are set up. Experts select the equipment required to the modular system of health emergency equipment, and the expert opinion column set up for experts to propose modification opinions.

##### 2.2.4.3 Consulting process

The questionnaire was sent to the experts via e-mail. After each round of consultation, the functional units of the equipment with a mean rationality (*M*) < 3.5 or a coefficient of variation (*CV*) > 0.25 were deleted or modified in accordance with the experts' opinions. The support rate (number of people agreeing/total number of people) of each piece of equipment was calculated, and equipment with a support rate < 0.8 was deleted or modified in accordance with the experts' opinions. The modified results were sent to the experts for the second round of consultation. Through the two rounds of expert consultation, the experts' opinions were harmonized and the modularized system of health emergency equipment for bioterrorism events in major public places was determined.

##### 2.2.4.4 Statistical analysis

The data were analyzed using the Excel 2023 and IBM Statistical Package for Social Sciences software version 26 to calculate the indicators of expert advice. The coefficients of the experts' positive responses were determined by calculating the response rate to the questionnaires (32, 33). The expert authority coefficient (*Cr*) is calculated to reflect the expert's cognition of the survey content, which is determined by two factors: the basis of expert judgment (*Ca*) and familiarity with the question (*Cs*) as *Cr* = (*Ca*+*Cs*)/2. The *Cr* value fluctuates between 0 and 1. The higher the expert authority coefficient is, the more reliable the results of the expert consultation. It is generally believed that when *Cr* ≥ 0.70, expert authority is high (34). The degree of expert coordination was reflected by calculating the coefficient of variation (*CV*) and Kendall′s *W*. The smaller the coefficient of variation (*CV*), the greater the degree of expert coordination. It is usually suggested that a *CV* < 0.25 is a good indicator (35). Kendall′s *W* was used to test the consistency of the experts' scoring results. *W* ranges from 0 to 1. The larger the value of *W* is, the greater the degree of coordination of the experts. The *p*-value of Kendall's *W* was statistically significant (*p* < 0.05) (36). Expert opinion is reflected by calculating the support rate of each equipment.

## 3 Results

### 3.1 Equipment function requirements analysis

In terms of the environment in which a bioterrorism event occurs, densely populated areas of activity, such as train stations, airports, subways, and other major public places, are prone to bioterrorist attacks. Such places are densely populated and have frequent contact with each other, and bioterrorism agents can spread rapidly and proliferate through close contact with the crowd, rapidly causing social panic and adverse effects (37). This situation requires that health emergency equipment be standardized, systematized, and modularized so that it can arrive quickly at the scene of the events with the health emergency team to carry out prevention, control, and disposal work. On the one hand, rapid detection and testing of bioterrorism agents should be conducted, and the types and pathways of the spread of bioterrorism agents should be determined. On the other hand, the spread of pathogenic factors should be interrupted to prevent further expansion of affected places and people, to isolate exposed people and to protect personnel at the scene to reduce the risk of spreading bioterrorism agents and a negative social impact. Bioterrorism event is likely to cause panic among the crowd, which may lead to crowding and stampedes, as well as injuries such as broken bones, ruptured internal organs, suffocation, and even death (38). Therefore, there is also a need to provide routine emergency medical treatment to those injured due to panic.

Public places are generally located in the center of the city, where nearby health emergency resources are relatively complete. For injured individuals and those confirmed infected with bioterrorism agents, stabilize their vital signs first, then rapidly transfer them to rear hospitals for further isolation and treatment (39, 40). Bioterrorism agents can be released through aerosols, biological vectors, contaminated food, and water. Health emergency personnel therefore need to collect samples of aerosols, vectors, food, and water at the scene for rapid testing to identify the type of bioterrorism agents. Because bioterrorism agents invade the human body mainly through respiratory inhalation, gastrointestinal tract ingestion, skin contact even conjunctival contamination, health emergency personnel, and exposed people need to be decontaminate and wear protective equipment to isolate them from bioterrorism agents (41, 42).

From the perspective of on-site personnel needs, there are survival needs and task needs. With regard to survival needs, health emergency personnel need protection due to direct or indirect exposure to bioterrorism agents during events handling. Exposed personnel need decontamination when leaving the scene after triage, and they need protection by vaccination or prophylactic drugs when entering the isolation area. From the perspective of task demand, natural disasters, accidents, and other health emergencies focus on emergency medical treatment of the injured. Due to the variety and toxicity of bioterrorism agents, they can cause disease or even death (43, 44), health emergency personnel focus on the rapid detection of bioterrorism agents on the scene and preventing their spread, such as disinfecting indoor and outdoor environments and trapping and killing vectors such as mosquitoes, insects, and rodents.

With regard to the methods and means of bioterrorism emergencies, according to the *General Emergency Response Plan for National Public Emergencies* (13), *Emergency Regulations for Public Health Emergencies* (14), as well as previous cases and practices (45, 46), the methods to address bioterrorism health emergencies at the scene can be summarized into the following six aspects. (1) After the emergency plan is launched, the relevant government departments where the emergency occurs shall, in accordance with the responsibilities and requirements stipulated in the emergency plan, obey the unified command of the emergency response headquarters, immediately arrive at their prescribed posts and take relevant control measures. (2) Epidemiological investigation should be conducted. After personnel from the centers for disease control and prevention arrive at the scene, epidemiological investigation plans and programs should be formulated as soon as possible. Local professional and technical personnel should conduct investigations and analyses of the incidence and distribution characteristics of the population affected by the emergency in accordance with plans and programs as well as targeted prevention and control measures. (3) Laboratory testing should be performed. Professional and technical institutions designated by the Chinese Center for Disease Control and Prevention and provincial disease prevention and control institutions, with the cooperation of local professional institutions, should take sufficient specimens according to the relevant technical specifications and send them to the provincial and national emergency response function network for laboratory testing to determine the cause of disease. The professional and technical institutions designated by the competent health administrative department of the State Council or other relevant departments have the right to enter the scene of an emergency to conduct investigations, sampling, technical analysis, and testing. (4) With regard to on-site rescue, after receiving rescue instructions, the medical and health rescue emergency team should rush to the scene and conduct medical and health rescue work according to the situation. In the process of implementing medical and health rescue, it is necessary to not only actively provide treatment but also pay attention to self-protection to ensure safety. (5) Transfer of the injured should be performed. The medical and health rescue emergency team arriving at the scene should quickly transport the injured out of the danger area in line with the principle of “saving lives before treating injuries, saving severe injuries before saving light” to carry out work and triage in accordance with international unified standards. (6) Patients with infectious diseases and those suspected to have infectious diseases should be isolated, observed and treated on the spot. Timely measures such as emergency vaccination, prophylactic medication and group protection should be taken for vulnerable groups.

In summary, on-site health emergencies involving bioterrorism in major public places have focused on the rapid investigation and inspection of bioterrorism agents, isolation, and decontamination to prevent the spread of bioterrorism agents, the protection of on-site disposal personnel and exposed personnel, and the treatment of injuries caused by trampling and crushing. Therefore, the functional needs of equipment are concentrated in five aspects: investigation, inspection, decontamination, protection, and treatment. Further analysis of the functional classification of each aspect shows that health emergency equipment should have 32 functions, as shown in Table 1, including command and communication, aerosol monitoring, guarantee flow adjustment, sampling, field detection, sample storage, environmental decontamination, personnel decontamination, vector killing, physical protection, medical protection, first aid, and evacuation.

**Table 1:** Preliminary program for a modularized system of health emergency equipment.

**Modularized system of health emergency equipment**			
**Level 1 Modules**	**Level 2 Modules**	**Level 3 Functional units**	**Equipment**
Investigation	Environmental monitoring	Aerosol monitoring	Real-time bioaerosol monitoring equipment, bioaerosol alarm system, biocapture device, handheld biological warfare agent detector, hand-held real-time infectious disease monitoring, and early warning equipment, air monitor, biological detectors/fully automated biological detectors/remote biological monitoring systems
	Epidemiologic investigation and analysis	Epidemiologic investigation and analysis	The portable site investigation and intelligent analysis system, portable field investigation and analysis system, comprehensive assessment and analysis system for infectious disease epidemics, and infectious disease surveillance, early warning and simulation technology platform
Detection	Body sampling	Patient sampling	Sampling robot, portable patient specimen collection kits, vomit specimen collection containers, specimen collection containers such as spatula tweezers, throat swabs, and their sealed containers, blood, urine, and stool specimen collection containers, tissue specimen collection containers
	Environmental sampling	Vector sampling	Vector sampling kits, animal sample collection bags, mosquito and egg traps, insect nets, rodent traps, rodent cages (boxes)
		Air microbial sampling	Bioaerosol samplers, air samplers (sampling pumps, collection devices)/gas samplers/air microbial samplers
		Soil microbial sampling	Soil microbial samplers, soil sample collection bags (bottles)
		Water microbial sampling	Microbiological samplers for water media, test tubes (bottles) for water samples/water quality samplers, portable planktonic bacteria samplers, deep well samplers
		Food microbial sampling	Residue container, suspicious food sampling container
	Sample storage	Sample storage	Refrigerator, ultra-low temperature refrigerator, temperature-controlled refrigerator, biosafety transfer case, insulated transportation boxes, multifunctional on-site specimen preservation and evacuation box, specimen box, transportation boxes, auxiliary packaging materials, liquid nitrogen tank, sample preservation box
	Sample detection	Molecular biological detection	Real-time fluorescence quantitative PCR instrument, gel imaging analysis system, nucleic acid rapid extractor, multi-channel nucleic acid fluorescence quantitative detection device, vehicle-mounted integrated nucleic acid rapid detection system, based on the principle of surface plasma resonance of pathogenic microorganisms biological portable on-site rapid detection system, hand-held digitization of real-time fluorescence PCR detector, portable real-time fluorescence quantitative constant-temperature nucleic acid amplification detector, portable ring-mediated isothermal amplifier (LAMP), portable cross-primer temperature amplifier
		Immunological testing	Vehicle-mounted multi-channel immuno rapid test system, portable piezoelectric sensor, visualized biochip high-throughput screening system, portable digital biosensing detection system, enzyme label analyzer, portable turbidimeter, portable digital colloidal gold-UPT dual mode detection module, fully automated immuno fluorescence coded rapid test system, portable chemiluminescence ELISA detector, portable time-resolved fluorescence imager
		Physicochemical testing	Food physical and chemical test chamber, water physical and chemical test chamber, air physical and chemical sampling chamber, electrophoresis system, ATP fluorometer, flow cytometer
		Microbiology detection	Water microbiology test kit, food microbiology test kit, animal infectious disease field test kit
		Blood and urine testing	Fully automated urine organic fraction analyzer, bloodborne pathogens rapid test kit, blood biochemistry analyzer
		General purpose detection	Portable biosafety cabinet, inverted fluorescence microscope, pipette, optical microscope, chromatography-mass spectrometer, electronic balance, glove box biocontainer, ultra-clean workbench, ultraviolet spectrophotometer, transport medium, liquid nitrogen cryopreservation box, general refrigerator, medical refrigerator, microscope, centrifuge, coagulation analyzer, biochemistry analyzer, blood counter, blood grouping test equipment, test equipment resupply kit, pathogen microbial detection vehicle, hand-held biological agent detectors, field bioassay kits, seismic advanced pathogen recognition instrument, biological rapid detection system
Decontamination	Decontamination	Personnel decontamination	Manual disinfectors, personal disinfection kits, skin sterilization kit, casualty decontamination units, inflatable decontamination tents, portable disinfection kits, decontamination systems
		Sterilization	Ethylene oxide sterilization chambers, autoclave sterilization chamber
		Equipment decontamination	Decontamination fluid preparation systems, decontamination carts, transportable decontamination systems
	Environmental disinfection	Indoor environmental disinfection	Microparticle sprayers, portable smoke sprayers, backpack motorized sprayers, backpack-type ultra-low-volume aerosol dispenser, hand-push type ULV sprayer, vehicle mounted ultra-low volume sprayers, vaporized hydrogen peroxide generators, gas chlorine dioxide disinfection units, compressed air foam backpacks, air foam decontamination equipment
		Outdoor environment disinfection	Turbo spray decontaminators, cart-mounted air foam decontamination equipment, trolley-type decontamination equipment, lightweight decontamination systems, ULV sprayers, electric/fuel-fired sprayers, hot or cold foggers, powder sprayers
	Vector extermination	Mosquitoes and insects extermination	Backpack insect sucker, sticky fly strip, sticky roach box, fly trap, mosquito lamp, electric mosquito absorber
		Rodent extermination	Sticky mouse boards/mouse traps
Protection and immunization	Protection	Individual protection	Personal protective clothing, half-body positive pressure suits, protective masks, lightweight full-body positive pressure suits, positive pressure protective hoods, medical protective masks, powered air supply respiratory protectio, air-carrying respiratory protectors, disposable medical suits, protective eyewear/eye protection/goggles, latex gloves, protective boots, protective footwear, biologically-specific protective clothing
		Collective protection	High-efficiency air filters, centrifugal fans, air drenches, oxygen regeneration devices, canisters, motorized sprayers, hand sprayers, tented soft protective equipment, NBC filtration systems, tent-type nuclear, chemical and biological collective protection and medical systems, collective protection and filtration equipment
	Antiepidemic	Immunization	On-site immunization boxes, vaccine storage and transportation boxes, epidemic prevention vehicles
		Preventive medicine	Portable medication kit [thymidine, dalfy, gamma-interferon, spectral antibiotics, mosquito barrier (avoidant)]
Medical ambulance	On-site first aid	On-site first aid	First aid backpacks, resuscitation (anti-shock) backpacks, primary debridement backpacks, defibrillators, infusion drug supply backpacks, infusion pumps
	Categorized evacuation	Triage	Triage marking
		Transit evacuation	Carrying backpacks (including folding stretcher, soft stretcher, negative pressure vacuum stretcher), neck braces (head immobilizers), negative pressure crash carts, medical kit sets
	Isolation	Isolation	Negative pressure tent/cabin, combination tent medical unit
	Integrated safeguards	Waste disposal	Decontamination waste liquid collection containers, non-hazardous medical waste collection devices, hazardous waste collection devices
		Command and communication	Digital camcorders, surveillance systems, mobile fax machines, walkie-talkies, GPS, command vehicles, laptop computer, multifunction printers, wireless LAN kit, hand-held amplifiers, portable projectors

### 3.2 Health emergency equipment screening

Based on the analysis results of the equipment function requirements, by analyzing the existing literature, data, and market research, the health emergency equipment is equipped according to the functions.

According to different types of bioterrorism agents and transmission routes, different kinds of monitoring, sampling, testing, decontamination, and protective equipment should be equipped. Taking the health emergency to the spread of *Bacillus anthracis* through airborne aerosols as an example, the sampling and testing should refer to the “Diagnosis for anthrax (WS283-2020)” (47), which requires the provision of environmental sampling equipment such as bioaerosol samplers, exposure and crowd-sampling equipment such as portable patient sample collection boxes, and handheld digital real-time fluorescent PCR detectors or anthrax rapid test kits (colloidal gold).

For decontamination of the environment, health emergency personnel, and exposed individuals, appropriate equipment—including personal disinfection kits, skin decontaminants, decontamination trucks, and backpack motorized sprayers and so on—should be selected based on environmental settings (indoor/outdoor) and the number of personnel involved; for the protection of personnel, according to the characteristics of the bioterrorism agents and the number of personnel, lightweight full-body positive-pressure suits, disposable medical suits, tent-type nuclear, chemical, biological and biological collective protection and medical systems can be equipped; When bioterrorism agents are transmitted through airborne aerosols, it is necessary to configure bio-aerosol real-time monitoring or alarm equipment. Command and communication, collective protection and other general-purpose categories can be equipped according to the configuration of the emergency epidemic prevention team, characteristics of biological bioterrorism agents, degree of impact, and other on-site task needs. Finally, as shown in Table 1, a preliminary plan of bioterrorism health emergency equipment system was established.

### 3.3 Preliminary plan for the modularized system of health emergency equipment

#### 3.3.1 Module divide

Equipment functional units with the same function or synergistic relationship are clustered through cluster analysis, and 32 equipment functional units are module divided. The functional units are digitized by DSM and 32 functional units are numbered in turn (N1–N32), as shown in Table 2.

**Table 2:** List of equipment functional units.

**Functional units' number**			
	**Functional units**		**Functional units**
N1	Aerosol monitoring	N17	Sterilization
N2	Epidemiologic investigation and analysis	N18	Equipment decontamination
N3	Patient sampling	N19	Indoor environmental disinfection
N4	Vector sampling	N20	Outdoor environmental disinfection
N5	Air microbial sampling	N21	Mosquitoes and insects extermination
N6	Soil microbial sampling	N22	Rodent extermination
N7	Water microbial sampling	N23	Individual protection
N8	Food microbial sampling	N24	Collective protection
N9	Sample storage	N25	Immunization
N10	Molecular biological detection	N26	Preventive medicine
N11	Immunological testing	N27	On-site first aid
N12	Physicochemical testing	N28	Triage
N13	Microbiology detection	N29	Transit evacuation
N14	Blood and urine testing	N30	Isolation
N15	General purpose detection	N31	Waste disposal
N16	Personnel decontamination	N32	Command and communication

The relationship matrix was determined according to the same or synergistic relationship between each equipment functional unit (see Supplementary Table S1); the clustering was then calculated using the Euclidean distance (see Supplementary Table S2).

#### 3.3.2 Module reorganization

The smaller the Euclidean distance *d* (i, j) means that the closer the elements i, j are, the higher the similarity is (48). Therefore, the equipment functional units of the same European distance result in the same row can be clustered into the same module. For example, in the first row, d4, d5, d6, d7, and d8 are all 2.4, and the N4 vector sampling unit, N5 air microbial sampling unit, N6 soil microbial sampling unit, N7 water microbial sampling unit, and N8 food microbial sampling unit can be clustered into the environmental sampling module. Finally, 32 equipment functional units can be combined into 15 modules. Continue reorganization by analyzing the hierarchical and sequential relationship of the 15 modules in terms of processes, tasks, and functions. Finally, a modularized system of bioterrorism health emergency equipment containing 5 primary modules, 15 secondary modules, and 15 tertiary modules was initially established.

#### 3.3.3 Revision and improvement of the modularized system of health emergency equipment

Fourteen experts with experience in biosecurity and health emergency response were invited from national and local centers for disease control and prevention, colleges and universities, research institutes, and relevant units of the military to participate in the expert consultation. Table 3 shows the demographics of the 14 experts.

**Table 3:** Demographics of the panelists (*n* = 14).

**Demographics of the panelists**			
**Categories**	**Characteristics**	**Number**	**Percentage(%)**
Gender	Male	9	64.3
	Female	5	35.7
Age	31–40 years	2	14.3
	41–50 years	7	50.0
	~51 years	5	35.7
Academic degree	Bachelor	3	21.4
	Master's	6	42.9
	Doctor	5	35.7
Professional title	Senior	14	100
Research fields	Biosecurity	3	21.4
	Biomedical engineering	5	35.7
	Health emergency management	6	42.9
Affiliation	University researchers	3	21.4
	National health emergency response team	5	35.7
	Military health emergency response team	2	14.3
	National and local CDC	4	28.6

#### 3.3.4 Expert positive coefficient and expert authority degree

The coefficients of the experts' positive responses were determined by calculating the response rate to the questionnaires. After two rounds of consultations, the response rates of the questionnaires were all 100% (14/14). Reasonable, scientific, and constructive advice was provided by the experts, and overall motivation was high. The expert authority coefficients of the two counseling questionnaires in this study were 0.83 and 0.88, respectively; therefore, the experts had high authority.

#### 3.3.5 The degree of expert coordination

The Kendall's *W* values for the 2 rounds of expert consultation were 0.226 and 0.258 (both *P* < 0.05), and the *W* values increased round by round with a better degree of expert coordination.

#### 3.3.6 Expert consultation results

In the first round of expert consultation on the modular system framework of health emergency equipment, 14 experts scored 5 first-level modules, 15 second-level modules and 32 third-level modules.

The results showed that the coefficient of variation (*CV*) for all modules was <0.25, and the mean rationality (*M*) was >3.5 for all modules except the “sterilization module” which was 2.4. Considering the comments made by the experts, the following modifications were made:

(1) The third-level module “patient sampling unit” was replaced with the “exposed population sampling unit” in accordance with the characteristics of a bioterrorist events;
(2) Due to the different specific equipment used, the second-level module “personnel decontamination” was split into “exposed personnel decontamination unit” and “emergency personnel decontamination unit.”
(3) According to the characteristics of bioterrorism agent, the site mostly focuses on decontamination, and few sterilization treatment, so the “sterilization unit” in the three-level module was deleted.

In round 2, the experts again scored the modules. The results showed that all modules had a mean rationality (*M*) of >3.5 and a coefficient of variation (*CV*) of < 0.25.

Opinions were sought on health emergency equipment through expert consultation, and revisions, additions or deletions were made based on expert opinion. There are two main criteria for experts to choose the equipment, one is whether the function of the equipment meets the needs of the on-site personnel (including health emergency personnel and the exposed personnel), and the other is whether it is suitable for the specific environment in a major public place of bioterrorism. Other standards, such as equipment logistics feasibility, cost, and interoperability, are not clearly specified in this study, but are considered according to the experts' own knowledge and experience. An expert support threshold of 80% was set, and the following revisions were made in light of the expert opinions:

(1) Thirty-five equipment items were deleted because of a noncompliant support rate, as shown in Table 4.
(2) The “ambulance” option was replaced with a “negative pressure ambulance” to avoid exposure to bioterrorism agents.
(3) “Biologically specific protective clothing” and “inspection injury classification system” were added.

**Table 4:** Consultation results of equipment.

**Substandard equipment**			
**Equipment**	**Support rate (%)**	**Equipment**	**Support rate (%)**
Biocapture device	78.6	Hand-push type ULV sprayer	71.4
Handheld biological warfare agent detector	78.6	Backpack-type ultra-low-volume aerosol dispenser	71.4
Biological detectors/fully automated biological detectors/remote biological monitoring systems	78.6	Vehicle mounted ultra-low volume sprayers	71.4
The portable site investigation and intelligent analysis system	71.4	Trolley-type decontamination equipment	78.6
Sampling robot	78.6	Air foam decontamination equip	78.6
Mosquito and egg traps	64.3	ULV sprayers	71.4
Refrigerator	78.6	Electric mosquito absorber	71.4
Ultra-low temperature refrigerator	78.6	Powered air supply respiratory protection	78.6
Insulated transportation boxes	71.4	Personal protective clothing	78.6
Transportation boxes	64.3	Hand sprayers	78.6
General refrigerator	64.3	Tented soft protective equip	78.6
Hand-held biological agent detectors	71.4	Seismic advanced pathogen recognition instrument	71.4
Field bioassay kits	71.4	Infusion pumps	71.4
NBC filtration systems	71.4	Laptop computer	78.6
Skin sterilization kit	78.6	Wireless LAN kit	64.3
Decontamination systems	78.6	Command vehicles	64.3
Autoclave sterilization chamber	42.8	Portable projectors	64.3
Ethylene oxide sterilization chambers	42.8		

After two rounds of expert consultation, the expert opinions agreed, and a modularized system of health emergency equipment in major public places, including 5 primary modules, 15 secondary modules, and 32 tertiary modules, equipped with 162 kinds of equipment, as shown in Table 5.

**Table 5:** Modularized system of on-site health emergency equipment for bioterrorism incidents in major public places.

**Modularized system of on-site health emergency equipment for bioterrorism incidents in major public places**			
**Level 1 Modules**	**Level 2 Modules**	**Level 3 Modules**	**Equipment**
Investigation	Environmental monitoring	Aerosol monitoring	Real-time bioaerosol monitoring equipment, bioaerosol alarm system, hand-held real-time infectious disease monitoring and early warning equipment, air monitors
	Epidemiologic investigation and analysis	Epidemiologic investigation and analysis	Portable field investigation and analysis system, comprehensive assessment and analysis system for infectious disease epidemics, and infectious disease surveillance, early warning and simulation technology platform
Detection	Body sampling	Exposed population sampling	Portable patient specimen collection kits, vomit specimen collection containers, specimen collection containers such as spatula tweezers, throat swabs and their sealed containers, blood, urine, and stool specimen collection containers, tissue specimen collection containers
	Environmental sampling	Vector sampling	Vector sampling kits, animal sample collection bags, insect nets, rodent traps, rodent cages (boxes)
		Air microbial sampling	Bioaerosol samplers, air samplers (sampling pumps/collection devices)/gas samplers/air microbial samplers
		Soil microbial sampling	Soil microbial samplers, soil sample collection bags (bottles)
		Water microbial sampling	Microbiological samplers for water media, test tubes (bottles) for water samples/water quality samplers, portable planktonic bacteria samplers, and deep well samplers
		Food microbial sampling	Residue container, suspicious food sampling container
	Sample storage	Sample storage	Temperature-controlled refrigerator, biosafety transfer case, multifunctional on-site specimen preservation and evacuation box, specimen box, auxiliary packaging materials, liquid nitrogen tank, sample preservation box
	Sample detection	Molecular biological detection	Real-time fluorescence quantitative PCR instrument, gel imaging analysis system, nucleic acid rapid extractor, multi-channel nucleic acid fluorescence quantitative detection device, vehicle-mounted integrated nucleic acid rapid detection system, based on the principle of surface plasma resonance of pathogenic microorganisms biological portable on-site rapid detection system, hand-held digitization of real-time fluorescence PCR detector, portable real-time fluorescence quantitative constant-temperature nucleic acid amplification detector, portable ring-mediated isothermal amplifier, portable cross-primer temperature amplifier
		Immunological testing	Vehicle-mounted multi-channel immuno rapid test system, portable piezoelectric sensor, visualized biochip high-throughput screening system, portable digital biosensing detection system, enzyme label analyzer, portable turbidimeter, portable digital colloidal gold-UPT dual mode detection module, fully automated immunofluorescence coded rapid test system, portable chemiluminescence ELISA detector, portable time-resolved fluorescence imager
		Physicochemical testing	Food physical and chemical test chamber, water physical and chemical test chamber, air physical and chemical sampling chamber, electrophoresis system, ATP eluorometer, flow cytometer
		Microbiology detection	Water microbiology test kit, food microbiology test kit, animal infectious disease field test kit
		Blood and urine testing	Fully automated urine organic fraction analyzer, bloodborne pathogens rapid test kit, blood biochemistry analyzer
		General purpose detection	Portable biosafety cabinet, inverted fluorescence microscope, pipette, optical microscope, chromatography-mass spectrometer, electronic balance, glove box biocontainer, ultra-clean workbench, ultraviolet spectrophotometer, transport medium, liquid nitrogen cryopreservation box, medical refrigerator, microscope, centrifuge, coagulation analyzer, biochemistry analyzer, blood counter, blood grouping test equipment, test equipment resupply kit, pathogen microbial detection vehicle, biological rapid detection system
Decontamination	Decontamination	Exposed personnel decontamination	Manual disinfectors, personal disinfection kits, casualty decontamination units, inflatable decontamination tents, portable disinfection kits
		Emergency personnel decontamination	Decontamination racks, skin decontaminants
		Equipment decontamination	Decontamination fluid preparation systems, decontamination carts, transportable decontamination systems
	Environmental disinfection	Indoor environmental disinfection	Microparticle sprayers, portable smoke sprayers, backpack motorized sprayers, vaporized hydrogen peroxide generators, gas chlorine dioxide disinfection units, compressed air foam backpacks
		Outdoor environment disinfection	Turbo spray decontaminators, cart-mounted air foam decontamination equipment, lightweight decontamination systems, electric/fuel-fired sprayers, hot or cold foggers, powder sprayers
	Vector extermination	Mosquitoes and insects extermination	Backpack insect sucker, sticky fly strip, sticky roach box, fly trap, mosquito lamp
		Rodent extermination	Sticky mouse boards/mouse traps
Protection and immunization	Protection	Individual protection	Half-body positive pressure suits, protective masks, lightweight full-body positive pressure suits, positive pressure protective hoods, medical protective masks, air-carrying respiratory protectors, disposable medical suits, protective eyewear/eye protection/goggles, latex gloves, protective boots, protective footwear, biologically-specific protective clothing
		Collective protection	High-efficiency air filters, centrifugal fans, air drenches, oxygen regeneration devices, canisters, motorized sprayers, tent-type nuclear, chemical and biological collective protection and medical systems, collective protection and filtration equipment
	Antiepidemic	Immunization	On-site immunization boxes, vaccine storage and transportation boxes, epidemic prevention vehicles
		Preventive medicine	Portable medication kit [thymidine, dalfy, gamma-interferon, spectral antibiotics, mosquito barrier (avoidant)]
Medical ambulance	On-site first aid	On-site first aid	First aid backpacks, resuscitation (anti-shock) backpacks, primary debridement backpacks, defibrillators, infusion drug supply backpacks
	Categorized evacuation	Triage	Triage marking, triage system
		Transit evacuation	Carrying backpacks (including folding stretcher, soft stretcher, negative pressure vacuum stretcher), neck braces (head immobilizers), negative pressure crash carts, medical kit sets
	Isolation	Isolation	Negative pressure tent/cabin, combination tent medical unit
	Integrated safeguards	Waste disposal	Decontamination waste liquid collection containers, non-hazardous medical waste collection devices, hazardous waste collection devices
		Command and communication	Digital camcorders, surveillance systems, mobile fax machines, walkie-talkies, GPS, multifunction printers, hand-held amplifiers

## 4 Discussion

Health emergency equipment is the material basis for the smooth implementation of emergency response work, and its specialization, standardization, and modularization directly determine the success or failure of health emergency response work. Currently, China has the *Health Emergency Response Team Equipment Reference Catalog (Trial)* (5), which standardizes the five categories of equipment for health emergency teams, namely, medical rescue, infectious disease control, poison disposal, management of nuclear and radiological injuries, and team security. However, the equipment and systematization of health emergency equipment for bioterrorism events have yet to be perfected.

This study systematically analyzed the equipment functional requirements from three aspects: the environment in which bioterrorism events occur, the survival and task requirements of personnel at the scene, and the methods of emergency response. The results show that the bioterrorism site health emergency response work focuses on the rapid detection and testing of bioterrorism agents, the protection of on-site disposal personnel and exposed personnel, and the isolation and decontamination measures taken to prevent the spread of bioterrorism agents, so the functional needs of the on-site disposal equipment are concentrated in the on-site environmental monitoring, sample sampling and monitoring, and the protection of personnel and equipment, environmental decontamination and other aspects (49–52). This is different from the natural disasters, accidents, and disasters and other events of health emergency disposal focus on the configuration of bandaging, hemostasis, immobilization, and other on-site emergency medical treatment equipment. However, considering the large number of people in major public places and the fact that a bioterrorism incident is very likely to cause panic among the crowd and lead to other accidents such as stampede, we have deliberately added the on-site first aid equipment module and the transportation and evacuation module to ensure that the injured can be sent to the hospital in a timely manner. Although there are sufficient medical resources in the vicinity of major public places, in consideration of the fact that there may be bioterrorism agents that are highly contagious and have a long incubation period, as well as the large number of people exposed at the scene, we have set up a collective protection unit to prevent and isolate the crowd.

It should be noted that the modular system of health emergency equipment constructed in this study is used for the health emergency of the injured and exposed people at the scene of a bioterrorism events, so the equipment that can decontaminate, protect, isolate, and quarantine the people at the scene has been selected. After the health emergency response team swiftly arrives at the scene, it will quickly seal off the area, protect, and decontaminate the personnel and vehicles entering and leaving the scene; protect, isolate, and quarantine the exposed people; protect, isolate, and transfer the confirmed infected people and the injured to the designated medical institutions. For those people who have leave the site because the health emergency response team did not arrive in time to take measures, we recommend that nearby emergency medical institutions be prepared to respond quickly to large groups of people who may arrive unexpectedly and who are not sure if they are infected. Emergency medical institutions should take rapid action to decontaminate, isolate, and quarantine personnel to reduce the risk of transmission of bioterrorism agents.

Based on the preliminary modularized system of health emergency equipment for bioterrorism events, this study adopts the Delphi expert consultation method to revise and improve. In terms of expert selection, this study invited 14 senior experts in the fields of biosafety, biomedical engineering, and health emergency management, 79% of whom have master's degree or above. The consultation results show that the positive coefficient of experts is 100%, higher than 70%, and the authority coefficient of experts is 0.83 and 0.88 respectively, both higher than 0.7 (34). It can be seen that the overall enthusiasm and authority of experts are high, which ensures the reliability of the expert consultation results. In the preliminary scheme of equipment modularized system, variation coefficient for all modules were <0.25, expert opinions were concentrated (35), Kendall's *W* was 0.226 and 0.258 respectively (*P* < 0.001), and *W* value increased round by round, indicating that the degree of expert coordination is good (36). According to the threshold of expert support and the opinions of experts, the equipment in the preliminary program was deleted and modified, and after 2 rounds of expert consultation, the opinions of experts converged, and the modularized system was finally formed, which contains 5 first-level modules, 15 second-level modules, 32 third-level modules, and 162 types of equipment for the on-site bioterrorist events in major public places.

### 4.1 Strengths

The modularized design of the equipment carried out in this study for bioterrorism health emergency makes the health emergency equipment highly flexible, which can be quickly combined and configured according to different emergency response scenarios and needs, and the speed of health emergency response can be improved by selecting the required modules according to the actual situation; Modularized design can improve the ability of collaborative operation. When responding to a bioterrorism incident, multi-departmental collaborative operation is required, and each department can select the corresponding module according to its work needs to realize the sharing and collaborative use of equipment in order to improve the efficiency of health emergency response; Modularized design makes it easy to expand and upgrade the equipment system, just add new modules or replace the old ones to meet the new requirements. For example, when a new bioterrorism agent emerges, new detection equipment, and vaccines can be developed and integrated into the existing modularized system of equipment to form a new modular system; Modularized design makes each health emergency equipment module easy to maintain and manage, a failure of a piece of equipment or module will not affect the overall operation, repair of faulty equipment or modules is sufficient.

### 4.2 Limitations

Due to the subjectivity of the Delphi method, the results obtained in this study have certain limitations, and the equipment modularized system needs to be further revised and improved, practiced and verified in subsequent empirical studies. Professional training and exercises can be organized for professionals or health emergency teams, through which the effectiveness and feasibility of the modular system of equipment can be tested, problems and deficiencies can be found, and timely improvements and optimization can be made. Furthermore, although we have considered the authority and representativeness of the experts as much as possible, a small panel size of 14 experts can reduce the diversity of perspectives and lead to narrower consensus, follow-up studies should consider this issue and select an appropriate number of experts for consultation. The modularized system of bioterrorism health emergency equipment only focuses on the function of the equipment when configuring the equipment, but does not deeply consider the problems such as the equipment cost constraints, which is not conducive to the application and practice of the modularized system of equipment, and it is necessary to continue to update this equipment system in the future. And the modularized system of equipment constructed in this study is only a part of the study of the bioterrorism health emergency equipment system, and in the future, it is necessary to carry out the allocation of the number of equipment, the assessment of the protection capability and simulation for the modularized system in order to further validate its scientificity and practicality.

## 5 Conclusion

In summary, in view of the characteristics of multiple types of equipment and strong specialization of the equipment required for on-site disposal of health emergency to bioterrorism events in major public places, this study corresponds the bioterrorism on-site scenarios to the functional requirements of the equipment through the scenario analysis, and the functional units of the equipment integrated are pertinent and reasonable; The module division using DSM is practical; Moreover, the equipment modularization system established by combining national standards for the provision of health emergency equipment, literature, market research and expert opinions is scientific and practical in nature. This study provides a scientific and feasible modularized system scheme of equipment for the on-site health emergency teams of bioterrorism events in major public places, which can improve the on-site disposal capacity and efficiency of health emergency personnel, and is of great significance to effectively respond to bioterrorism events.

## Data Availability

The original contributions presented in the study are included in the article/Supplementary material, further inquiries can be directed to the corresponding author.
