# Supplementary material for: Design of a public health emergency equipment modularized system for bioterrorism events in major public places based on scenario analysis, literature review, cluster analysis, and Delphi consultation
Source: Front Public Health. 2025 Apr 17;13:1513319. doi: 10.3389/fpubh.2025.1513319 (PMC12043893; doi:10.3389/fpubh.2025.1513319)
Supplement: Supplementary file 1 [file Data_Sheet_1.pdf]

Supplementary Material

Table S1 Relationship matrix of the equipment functional units

	**Relationship matrix**																															
	**N1**	**N2**	**N3**	**N4**	**N5**	**N6**	**N7**	**N8**	**N9**	**N10**	**N11**	**N12**	**N13**	**N14**	**N15**	**N16**	**N17**	**N18**	**N19**	**N20**	**N21**	**N22**	**N23**	**N24**	**N25**	**N26**	**N27**	**N28**	**N29**	**N30**	**N31**	**N32**
**N1**	1	0	0	0	0	0	0	0	0	0	0	0	0	0	0	0	0	0	0	0	0	0	0	0	0	0	0	0	0	0	0	0
**N2**	0	1	0	0	0	0	0	0	0	0	0	0	0	0	0	0	0	0	0	0	0	0	0	0	0	0	0	0	0	0	0	0
**N3**	0	0	1	0	0	0	0	0	0	0	0	0	0	0	0	0	0	0	0	0	0	0	0	0	0	0	0	0	0	0	0	0
**N4**	0	0	0	1	1	1	1	1	0	0	0	0	0	0	0	0	0	0	0	0	0	0	0	0	0	0	0	0	0	0	0	0
**N5**	0	0	0	1	1	1	1	1	0	0	0	0	0	0	0	0	0	0	0	0	0	0	0	0	0	0	0	0	0	0	0	0
**N6**	0	0	0	1	1	1	1	1	0	0	0	0	0	0	0	0	0	0	0	0	0	0	0	0	0	0	0	0	0	0	0	0
**N7**	0	0	0	1	1	1	1	1	0	0	0	0	0	0	0	0	0	0	0	0	0	0	0	0	0	0	0	0	0	0	0	0
**N8**	0	0	0	1	1	1	1	1	0	0	0	0	0	0	0	0	0	0	0	0	0	0	0	0	0	0	0	0	0	0	0	0
**N9**	0	0	0	0	0	0	0	0	1	0	0	0	0	0	0	0	0	0	0	0	0	0	0	0	0	0	0	0	0	0	0	0

**N10**	0	0	0	0	0	0	0	0	0	1	1	1	1	1	1	0	0	0	0	0	0	0	0	0	0	0	0	0	0	0	0	0
**N11**	0	0	0	0	0	0	0	0	0	1	1	1	1	1	1	0	0	0	0	0	0	0	0	0	0	0	0	0	0	0	0	0
**N12**	0	0	0	0	0	0	0	0	0	1	1	1	1	1	1	0	0	0	0	0	0	0	0	0	0	0	0	0	0	0	0	0
**N13**	0	0	0	0	0	0	0	0	0	1	1	1	1	1	1	0	0	0	0	0	0	0	0	0	0	0	0	0	0	0	0	0
**N14**	0	0	0	0	0	0	0	0	0	1	1	1	1	1	1	0	0	0	0	0	0	0	0	0	0	0	0	0	0	0	0	0
**N 15**	0	0	0	0	0	0	0	0	0	1	1	1	1	1	1	0	0	0	0	0	0	0	0	0	0	0	0	0	0	0	0	0
**N16**	0	0	0	0	0	0	0	0	0	0	0	0	0	0	0	1	1	1	0	0	0	0	0	0	0	0	0	0	0	0	0	0
**N17**	0	0	0	0	0	0	0	0	0	0	0	0	0	0	0	1	1	1	0	0	0	0	0	0	0	0	0	0	0	0	0	0
**N18**	0	0	0	0	0	0	0	0	0	0	0	0	0	0	0	1	1	1	0	0	0	0	0	0	0	0	0	0	0	0	0	0
**N19**	0	0	0	0	0	0	0	0	0	0	0	0	0	0	0	0	0	0	1	1	0	0	0	0	0	0	0	0	0	0	0	0
**N20**	0	0	0	0	0	0	0	0	0	0	0	0	0	0	0	0	0	0	1	1	0	0	0	0	0	0	0	0	0	0	0	0
**N21**	0	0	0	0	0	0	0	0	0	0	0	0	0	0	0	0	0	0	0	0	1	1	0	0	0	0	0	0	0	0	0	0
**N22**	0	0	0	0	0	0	0	0	0	0	0	0	0	0	0	0	0	0	0	0	1	1	0	0	0	0	0	0	0	0	0	0
**N23**	0	0	0	0	0	0	0	0	0	0	0	0	0	0	0	0	0	0	0	0	0	0	1	1	0	0	0	0	0	0	0	0
**N24**	0	0	0	0	0	0	0	0	0	0	0	0	0	0	0	0	0	0	0	0	0	0	1	1	0	0	0	0	0	0	0	0

**N25**	0	0	0	0	0	0	0	0	0	0	0	0	0	0	0	0	0	0	0	0	0	0	0	0	1	1	0	0	0	0	0	0
**N26**	0	0	0	0	0	0	0	0	0	0	0	0	0	0	0	0	0	0	0	0	0	0	0	0	1	1	0	0	0	0	0	0
**N27**	0	0	0	0	0	0	0	0	0	0	0	0	0	0	0	0	0	0	0	0	0	0	0	0	0	0	1	0	0	0	0	0
**N28**	0	0	0	0	0	0	0	0	0	0	0	0	0	0	0	0	0	0	0	0	0	0	0	0	0	0	0	1	1	0	0	0
**N29**	0	0	0	0	0	0	0	0	0	0	0	0	0	0	0	0	0	0	0	0	0	0	0	0	0	0	0	1	1	0	0	0
**N30**	0	0	0	0	0	0	0	0	0	0	0	0	0	0	0	0	0	0	0	0	0	0	0	0	0	0	0	0	0	1	0	0
**N31**	0	0	0	0	0	0	0	0	0	0	0	0	0	0	0	0	0	0	0	0	0	0	0	0	0	0	0	0	0	0	1	1
**N32**	0	0	0	0	0	0	0	0	0	0	0	0	0	0	0	0	0	0	0	0	0	0	0	0	0	0	0	0	0	0	1	1

Table S2 The Euclidean Distance of Each unit

	Euclidean Distance																														
	**d2**	**d3**	**d4**	**d5**	**d6**	**d7**	**d8**	**d9**	**d 10**	**d 11**	**d 12**	**d 13**	**d 14**	**d 15**	**d 16**	**d 17**	**d 18**	**d 19**	**d 20**	**d 21**	**d 22**	**d 23**	**d 24**	**d 25**	**d 26**	**d 27**	**d 28**	**d 29**	**d 30**	**d 31**	**d 32**
**d1**	1.4	1.4	2.4	2.4	2.4	2.4	2.4	1.4	2.6	2.6	2.6	2.6	2.6	2.6	2.0	2.0	2.0	1.7	1.7	1.7	1.7	1.7	1.7	1.7	1.7	1.4	1.7	1.7	1.4	1.7	1.7
**d2**		1.4	2.4	2.4	2.4	2.4	2.4	1.4	2.6	2.6	2.6	2.6	2.6	2.6	2.0	2.0	2.0	1.7	1.7	1.7	1.7	1.7	1.7	1.7	1.7	1.4	1.7	1.7	1.4	1.7	1.7
**d3**			2.4	2.4	2.4	2.4	2.4	1.4	2.6	2.6	2.6	2.6	2.6	2.6	2.0	2.0	2.0	1.7	1.7	1.7	1.7	1.7	1.7	1.7	1.7	1.4	1.7	1.7	1.4	1.7	1.7
**d4**				0	0	0	0.0	2.4	3.3	3.3	3.3	3.3	3.3	3.3	2.8	2.8	2.8	2.6	2.6	2.6	2.6	2.6	2.6	2.6	2.6	2.4	2.6	2.6	2.4	2.6	2.6
**d5**					0	0	0.0	2.4	3.3	3.3	3.3	3.3	3.3	3.3	2.8	2.8	2.8	2.6	2.6	2.6	2.6	2.6	2.6	2.6	2.6	2.4	2.6	2.6	2.4	2.6	2.6
**d6**						0	0	2.4	3.3	3.3	3.3	3.3	3.3	3.3	2.8	2.8	2.8	2.6	2.6	2.6	2.6	2.6	2.6	2.6	2.6	2.4	2.6	2.6	2.4	2.6	2.6
**d7**							0	2.4	3.3	3.3	3.3	3.3	3.3	3.3	2.8	2.8	2.8	2.6	2.6	2.6	2.6	2.6	2.6	2.6	2.6	2.4	2.6	2.6	2.4	2.6	2.6
**d8**								2.4	3.3	3.3	3.3	3.3	3.3	3.3	2.8	2.8	2.8	2.6	2.6	2.6	2.6	2.6	2.6	2.6	2.6	2.4	2.6	2.6	2.4	2.6	2.6
**d9**									2.6	2.6	2.6	2.6	2.6	2.6	2.0	2.0	2.0	1.7	1.7	1.7	1.7	1.7	1.7	1.7	1.7	1.4	1.7	1.7	1.4	1.7	1.7
**d10**										0	0	0	0	0	3.0	3.0	3.0	2.8	2.8	2.8	2.8	2.8	2.8	2.8	2.8	2.6	2.8	2.8	2.6	2.8	2.8
**d11**											0	0	0	0	3.0	3.0	3.0	2.8	2.8	2.8	2.8	2.8	2.8	2.8	2.8	2.6	2.8	2.8	2.6	2.8	2.8
**d12**												0	0	0	3.0	3.0	3.0	2.8	2.8	2.8	2.8	2.8	2.8	2.8	2.8	2.6	2.8	2.8	2.6	2.8	2.8

**d13**	0	0	3.0	3.0	3.0	2.8	2.8	2.8	2.8	2.8	2.8	2.8	2.8	2.6	2.8	2.8	2.6	2.8	2.8
**d14**		0	3.0	3.0	3.0	2.8	2.8	2.8	2.8	2.8	2.8	2.8	2.8	2.6	2.8	2.8	2.6	2.8	2.8
**d15**			3.0	3.0	3.0	2.8	2.8	2.8	2.8	2.8	2.8	2.8	2.8	2.6	2.8	2.8	2.6	2.8	2.8
**d16**				0	0	2.2	2.2	2.2	2.2	2.2	2.2	2.2	2.2	2.0	2.2	2.2	2.0	2.2	2.2
**d17**					0	2.2	2.2	2.2	2.2	2.2	2.2	2.2	2.2	2.0	2.2	2.2	2.0	2.2	2.2
**d18**						2.2	2.2	2.2	2.2	2.2	2.2	2.2	2.2	2.0	2.2	2.2	2.0	2.2	2.2
**d19**							0	2.0	2.0	2.0	2.0	2.0	2.0	1.7	2.0	2.0	1.7	2.0	2.0
**d20**								2.0	2.0	2.0	2.0	2.0	2.0	1.7	2.0	2.0	1.7	2.0	2.0
**d21**									0	2.0	2.0	2.0	2.0	1.7	2.0	2.0	1.7	2.0	2.0
**d22**										2.0	2.0	2.0	2.0	1.7	2.0	2.0	1.7	2.0	2.0
**d23**											0	2.0	2.0	1.7	2.0	2.0	1.7	2.0	2.0
**d24**												2.0	2.0	1.7	2.0	2.0	1.7	2.0	2.0
**d25**													0	1.7	2.0	2.0	1.7	2.0	2.0
**d26**														1.7	2.0	2.0	1.7	2.0	2.0
**d27**															1.7	1.7	1.4	1.7	1.7
**d28**																0	1.7	2.0	2.0
**d29**																	1.7	2.0	2.0

**d30**	1.7	1.7
**d31**		0
